# Effects of Various Allelic Combinations of Starch Biosynthetic Genes on the Properties of Endosperm Starch in Rice

**DOI:** 10.1186/s12284-022-00570-8

**Published:** 2022-04-19

**Authors:** Naoko Fujita, Satoko Miura, Naoko Crofts

**Affiliations:** Department of Biological Production, Akita Prefectural University, Akita, 010-0195 Japan

**Keywords:** Amylopectin, Amylose, Backcrossing, Endosperm, *Indica* rice, *Japonica* rice, Starch biosynthesis, Starch branching enzyme, Starch debranching enzyme, Starch synthase

## Abstract

Rice endosperm accumulates large amounts of photosynthetic products as insoluble starch within amyloplasts by properly arranging structured, highly branched, large amylopectin molecules, thus avoiding osmotic imbalance. The amount and characteristics of starch directly influence the yield and quality of rice grains, which in turn influence their application and market value. Therefore, understanding how various allelic combinations of starch biosynthetic genes, with different expression levels, affect starch properties is important for the identification of targets for breeding new rice cultivars. Research over the past few decades has revealed the spatiotemporal expression patterns and allelic variants of starch biosynthetic genes, and enhanced our understanding of the specific roles and compensatory functions of individual isozymes of starch biosynthetic enzymes through biochemical analyses of purified enzymes and characterization of *japonica* rice mutants lacking these enzymes. Furthermore, it has been shown that starch biosynthetic enzymes can mutually and synergistically increase their activities by forming protein complexes. This review focuses on the more recent discoveries made in the last several years. Generation of single and double mutants and/or high-level expression of specific starch synthases (SSs) allowed us to better understand how the starch granule morphology is determined; how the complete absence of SSIIa affects starch structure; why the rice endosperm stores insoluble starch rather than soluble phytoglycogen; how to elevate amylose and resistant starch (RS) content to improve health benefits; and how SS isozymes mutually complement their activities. The introduction of active-type *SSIIa* and/or high-expression type *GBSSI* into *ss3a ss4b*, *isa1*, *be2b*, and *ss3a be2b japonica* rice mutants, with unique starch properties, and analyses of their starch properties are summarized in this review. High-level accumulation of RS is often accompanied by a reduction in grain yield as a trade-off. Backcrossing rice mutants with a high-yielding elite rice cultivar enabled the improvement of agricultural traits, while maintaining high RS levels. Designing starch structures for additional values, breeding and cultivating to increase yield will enable the development of a new type of rice starch that can be used in a wide variety of applications, and that can contribute to food and agricultural industries in the near future.

## Background

Starch (α-glucan), which is synthesized mainly from photosynthetic products, is deposited in the storage tissues of plants and represents the most important source of carbohydrates in the human diet. Starch is a gigantic polymer of glucose molecules connected by α-1,4- and α-1,6-glucosidc bonds. The major component of starch is amylopectin, a highly branched structure, while that remaining is composed of amylose, which is essentially a linear polymer. The structure of starch, as described above, has been extensively studied at the molecular level; however, the higher order structure of starch has yet to be fully understood. To date, the higher order structure of starch has been postulated by two models and is currently under debate: the Cluster Model (Nikuni 1969; French 1972; Hizukuri 1986) has been proposed by many researchers, and the Backbone Model (Bertoft et al. 2012) is currently being challenged (Crini et al. 2021; Nakamura and Kainuma 2021). 

Starch biosynthesis involves at least four classes of enzymes (Smith et al. 1997): ADP-glucose pyrophosphorylase (AGPase), which provides glucose moiety as a substrate; starch synthases (SSs), which use the substrate to elongate α-1,4-linked linear glucose chains; starch branching enzymes (BEs), which create α-1,6-linked branches; and starch debranching enzymes (DBEs), which trim off improper branches (Nakamura 2002). Additional enzymes are also involved in starch biosynthesis: starch phosphorylase, which functions in the initiation step of starch biosynthesis (Satoh et al. 2008; Nakamura et al. 2012); disproportionating enzyme, which transfers α-1,4-linked glucans (Colleoni et al. 1999a, b); and Protein Targeting to Starch (PTST), which guides starch biosynthetic enzymes to the starch granules (Seung et al. 2018). The function of each starch biosynthetic enzyme was elucidated in the 1960s to 1980s using biochemical approaches, and the DNA sequence of the corresponding genes was determined in major crops in the 1990s. Our understanding of the mechanisms of starch biosynthesis rapidly improved after the 2000s because of the isolation, identification, and characterization of mutants lacking starch biosynthetic enzymes in major crops such as maize, rice, wheat, barley, potato, and beans, in addition to model organisms such as *Arabidopsis* and *Chlamydomonas.* Our research group revealed the functions of each starch biosynthetic enzyme by exhaustively isolating *japonica* rice mutants lacking these enzymes and by comparing the characteristics of these mutants with those of the wild-type in detail. The results of our studies served as the basis for the model of starch biosynthesis (Nakamura 2002; Fujita 2014).

The abovementioned rice mutants not only contributed to the discovery of starch biosynthesis mechanisms but also displayed great potential for expanding the use of rice starch in various applications. The starch accumulated in some of these rice mutants exhibited unique physicochemical properties. For example, the texture of high amylose mutant rice *ss3a*, after cooking, is utterly different from that of wild-type *japonica* rice. The *be2b* mutants, which contain high levels of resistant starch (RS), are likely to be utilized as functional rice because the intake of RS is thought to promote human health. Greater variation in the properties of rice starch will likely expand its utilization in various food applications. In this review, the process of generating new rice lines, with unique and novel starch properties, by designing the starch structure, is explained based on accumulating evidence obtained through the analysis of single and double mutants, and by the introduction of high-expressing alleles of starch biosynthetic genes. Please refer to the book “Starch” on rice lines generated before 2015 (Fujita 2015). The present review focuses on rice lines generated after 2015, and describes the breeding of new promising rice cultivars and their potential application in the food industry.

## Methods

Mutant rice lines described in this article were isolated from the *N*-Methyl-*N*-nitrosourea (MNU)-treated populations of *japonica* rice cultivars (Kinmaze and Taichung 65) or from the *Tos17* insertional mutant panels of *japonica* rice cultivar, Nipponbare. Mutant lines were identified by western blot analysis of mature seed extracts to confirm the absence of specific proteins. The presence of single nucleotide polymorphisms (SNPs) was confirmed by PCR amplification of genomic DNA isolated from seedlings, followed by genotyping using cleaved amplified polymorphic sequence (CAPS) or derived cleaved amplified polymorphic sequence (dCAPS) markers. *SSIIa* and/or *GBSSI* genes derived from *indica* rice were selected by PCR using SNP-specific primers. Mutant lines lacking starch biosynthetic enzyme(s) were backcrossed with elite rice cultivars, ‘Akita 63’ or ‘Akita Komachi’, and homozygous BC_3_F_2_ plants were identified as described above. The selected homozygous plants were grown to maturity, and the seeds were used for extracting starch using the cold-alkaline method. The purified starch was gelatinized, debranched using *Pseudomonas* isoamylase, and separated by gel filtration chromatography using ToyoPearl HW55s connected in series to three ToyoPearl 50S columns to analyze the apparent amylose content. The amylopectin branch structure was analyzed by capillary electrophoresis, and starch granule morphology was observed by scanning electron microscopy.

## *SSIIa* Alleles

Most of the *indica* rice cultivars possess active-type SSIIa (wild-type), whereas typical *japonica* rice cultivars possess mutant SSIIa harboring three amino acid substitutions, thus exhibiting only 10% of SSIIa activity relative to *indica* rice (Nakamura et al. 2005). Low SSIIa activity in *japonica* rice leads to a reduction in amylopectin branches with degree of polymerization (DP) 13–24, and an increase in short amylopectin chains with DP ≤ 12 (Umemoto et al. 1999, 2002; Nakamura et al. 2005). These changes in amylopectin branch structure drastically affect the gelatinization temperature of starch (Noda et al. 1998), which is 5–10 °C lower in *japonica* rice than in *indica* rice (Nakamura et al. 2002). The effects of SSIIa absence on amylopectin branch structure have also been reported in other plant species such as maize (Zhang et al. 2004; Liu et al. 2012), wheat (Yamamori et al. 2000), barley (Morell et al. 2003), sweet potato (Katayama et al. 2002; Kitahara et al. 2005), and *Arabidopsis* (Zhang et al. 2008). Loss of SSIIa in maize (Takeda and Preiss 1993), wheat (Yamamori et al. 2000), barley (Morell et al. 2003), and *Arabidopsis* (Zhang et al. 2008) was accompanied by an increase in amylose content. However, unlike other plant species, accurate evaluation of the association between the increase in amylose content and the loss of SSIIa is not straight forward in rice. This is because the amylose biosynthesis gene *granule bound-starch synthase I* (*GBSSI*), which is located close to the *SSIIa* gene on chromosome 6, carries a SNP at the exon1–intron1 boundary in *japonica* rice, which reduces the amount of GBSSI protein, thus decreasing the amylose content in *japonica* rice compared with *indica* rice (Sano 1984).

**Fig. 1:**
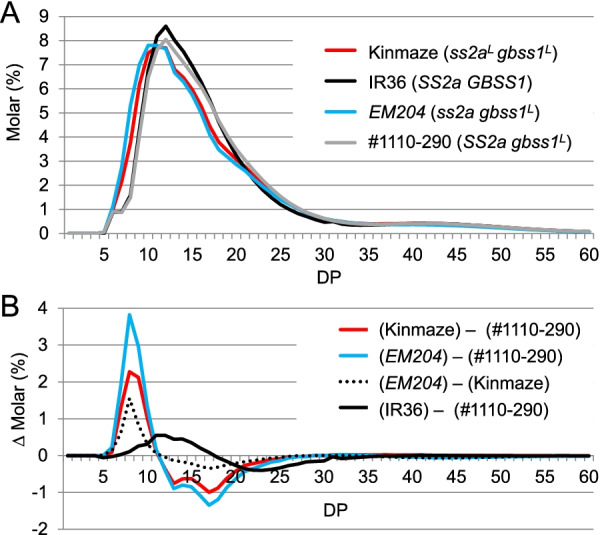
Analysis of the molecular structure of amylopectin by capillary electrophoresis. **A** Chain-length distribution patterns of amylopectin in mature rice endosperm. **B** Differential plots of Kinmaze (*japonica* rice; *ss2a*^*L*^* gbss1*^*L*^), EM204 (*ss2a* mutant; *ss2a gbss1*^*L*^), and line #1110-290 (expressing *indica*-type SSIIa; *SS2a gbss1*^*L*^). Each panel shows a typical representative data set of at least three replications. DP, degree of polymerization. Data were obtained from Miura et al. (2018)

We isolated a null mutant of the *SSIIa* gene, EM204, from the MNU mutant panel of the *japonica* rice cultivar Kinmaze (Miura et al. 2018). EM204 possesses a SNP at the end of the 5^th^ intron, resulting in the skipping of the 6th exon during translation, eventually producing a negligible amount of the truncated SSIIa protein lacking 15 amino acids (Miura et al. 2018). EM204 showed no SSIIa activity, higher amounts of short amylopectin chains (DP ≤ 12), and lower amounts of amylopectin branches with DP 13–24 compared with wild-type *japonica* rice, expressing SSIIa with low activity (Fig. 1; Miura et al. 2018). Comparison of amylose content between lines containing or lacking SSIIa under the same *japonica*-type *gbss1*^*L*^ allele revealed that loss of SSIIa led to a higher amylose content, as in other plant species (Miura et al. 2018).

Knocking down SSIIa from wild-type *japonica* rice, Nipponbare, by RNAi also did not show any increase in amylose content in endosperm starch. Its amylopectin structure, physicochemical properties of starch, and seed phenotype resembled those of EM204 (Butardo et al. 2020). *SSIIa* locus is known to be the same as *ALK* (Umemoto et al. 1999, 2002), and there are four known alleles, *ALK*^*a*^, *ALK*^*b*^, *ALK*^*c*^, *ALK*^*d*^, depending on the combinations of amino acid substitutions (Zhang et al. 2020; Chen et al. 2020). *ALK*^*c*^ encodes *indica*-type active SSIIa, whereas *ALK*^*a*^ encodes *japonica*-type SSIIa with Glu88Asp, Gly604Ser, Val737Met substitutions resulting in low activity and an increase in amylopectin short chains with DP < 12 (Nakamura et al. 2005; Zhang et al. 2020). *ALK*^*b*^ encodes *japonica*-type SSIIa with Glu88Asp, Gly604Ser, Leu781Phe substitutions, and analyses of near isogenic lines showed a further increase in amylopectin short chains with DP < 12 and decrease in gelatinization temperature compared to *ALK*^*a*^. In contrast, *ALK*^*d*^ with Glu to Asp substitution in Exon 1 of *ALK*^*c*^ showed a decrease in short amylopectin chains with DP < 12 and an increase in gelatinization temperature (Zhang et al. 2020). Given abovementioned starch structure characteristics of *SSIIa* alleles, it is no doubt that the strength of SSIIa activity determines the ratio of short amylopectin chains with DP < 12 and intermediate amylopectin chains with DP 13–24. These greatly influence physicochemical properties such as gelatinization temperature and retrogradation of starch. Particularly, the *ss2a* mutant or SSIIa knockdown rice lines could be utilized practically as a genetic material for preventing the retrogradation of starch, and for breeding rice that tastes excellent after cooking and that maintains its stickiness and softness after cooling.

## Ultra-High RS Content Rice

Rice and maize genomes encode three BE isozymes, BEI, BEIIa, and BEIIb, and single mutants of each *BE* gene have been isolated in *japonica* rice. *BEI* is expressed in both endosperm and vegetative tissues (Yamanouchi and Nakamura 1992). The phenotype of the *be1* single mutant generally resembles that of the wild-type, with minor differences in endosperm amylopectin structure; the amount of amylopectin branches with DP 12–21 and DP > 37 is slightly higher in *be1*, whereas that of DP < 10 is slightly increased, compared with the wild-type (Satoh et al. 2003). BEIIa is highly expressed in vegetative organs, and its absence does not affect the structure of endosperm starch (Nakamura 2002). By contrast, BEIIb is expressed exclusively in the endosperm, and *be2b* mutants exhibit a drastically different endosperm starch structure compared with the wild-type *japonica* rice: the amount of amylopectin chains with DP < 14 is much lower, while the amylose content is considerably higher in *be2b* mutants than in the wild-type (Nishi et al. 2001). Owing to its unique structure, starch in *be2b* mutants resists degradation by digestive enzymes, resulting in high RS content (Tsuiki et al. 2016; Miura et al. 2021). Knocking out BEIIb in *indica* rice by CRISPR/Cas9 approach gave similar effects on starch properties, and increased amylopectin branches with DP 6, 7 and > 25, decreased amylopectin chains with DP 9–24, increased gelatinization temperature by 6 °C, and increased amylose content by 7% (Tappiban et al. 2021).

RS is defined as the starch that is not easily digested by human digestive enzymes and therefore reaches the large intestine, where it prevents a sudden increase in postprandial glucose levels and improves the intestinal environment, thus promoting human health (Englyst et al. 1992; Nugent 2005; Matsuki 2010). RS contents were analyzed in various rice cultivars and mutants, including *indica* rice cultivars with high amylose content, single and double mutants of *ss3a* with high amylose content, and single and double mutants of *be2b* with high levels of long amylopectin chains. The results of the survey revealed that the RS contents of *be2b* mutants were an order of magnitude higher than those of other lines. The RS content of high amylose rice, such as *indica* rice and the *ss3a* single mutant, was only several times higher than that of wild-type *japonica* rice. Previously, amylose was considered to be one of the factors contributing to the RS content; however, our study clearly demonstrated that high levels of long amylopectin branches is the primary factor responsible for the increase in RS content (Tsuiki et al. 2016).

**Fig. 2:**
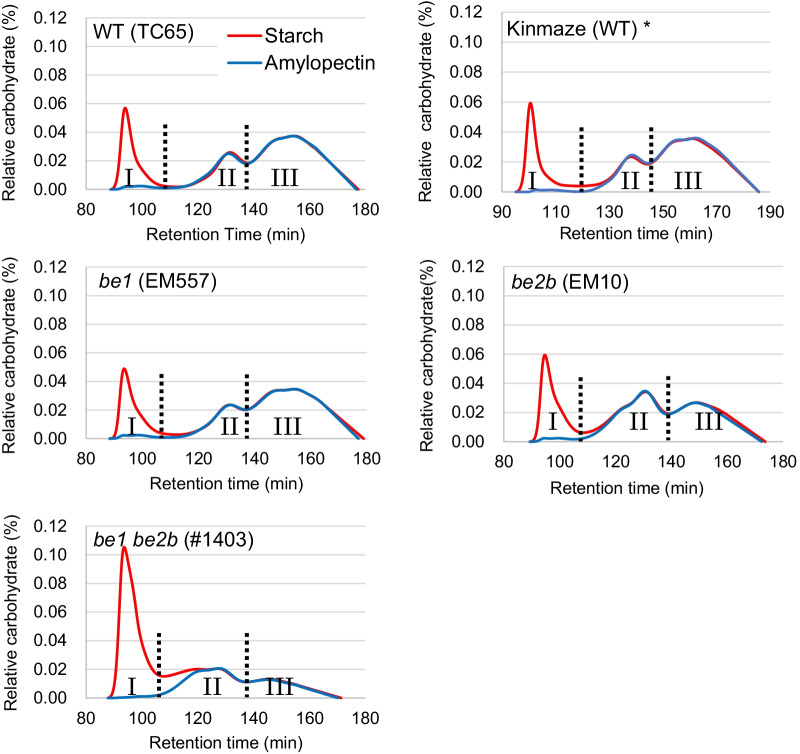
Elution profiles of isoamylase-treated debranched starch and amylopectin analyzed by gel filtration chromatography (ToyoPearl HW55S-50S × 3). Fraction I contains amylose or an extra-long chain of amylopectin. Fraction II contains long amylopectin chains. Fraction III contains short amylopectin chains. Red lines indicate the patterns obtained from starch, and blue lines indicate the patterns obtained from purified amylopectin. *Differences in retention time (RT) in Kinmaze were due to the different lot of the column. Data was obtained from Miura et al. (2021)

Compared with the *be2b* single mutant, the *be1 be2b* double mutant contained fewer amylopectin chains with DP 10–20 and more amylopectin chains with DP ≥ 21, and showed an amylose content of 52%, which was the highest in the entire rice lines (Fig. 2; Miura et al. 2021). Given its extreme starch composition, the *be1 be2b* double mutant showed an increase in the gelatinization temperature of starch by 7.5 °C, and in the RS content of its cooked rice (76%) by threefold, compared with the *be2b* single mutant. Further analysis demonstrated that RS extracted from the cooked rice of *be1 be2b* consisted of partially degraded amylose and long amylopectin branches. Although the *be1 be2b* double mutant lacked two major BE isozymes, its plant growth and fertility rate were not affected, and it showed rather higher seed weight than the *be2b* single mutant (Miura et al. 2021). Suppression of *BEI* and *BEIIb* expression in *japonica* rice using the transgenic approach has been reported previously (Zhu et al. 2012; Wang et al. 2017; Pan et al. 2018; Sawada et al. 2018); however, this was the first report of *BEI* and *BEIIb* suppression in non-transgenic rice. Further breeding of RS-rich rice lines with improved agricultural traits will provide valuable genetic material for producing functional foods in the near future.

## Introduction of *SS2a *from *Indica *Rice into *ss1 *and *ss3a*

Among all starch biosynthetic enzymes, SSs possess the largest numbers of isozymes. Among these isozymes, SSI, SSIIa, and SSIIIa play major roles in amylopectin biosynthesis in the endosperm. As described above, SSIIa found in *indica* rice is the highly active wild-type enzyme, whereas that found in *japonica* rice exhibits only 10% of the activity of *indica* SSIIa (Nakamura et al. 2005). Because of the low-level activity of SSIIa in *japonica* rice, the genotype of wild-type *japonica* rice is shown as *SS1 ss2a*^*L*^* SS3a*. *Japonica* rice mutants lacking SSI (Fujita et al. 2006) or SSIIIa (Fujita et al. 2007) have been isolated previously, both of which show no activity of the corresponding enzymes and different amylopectin structures relative to wild-type *japonica* rice. However, the *ss1* and *ss3a* mutants accumulate starch to levels similar to the wild-type, which implies that other SS isozymes complement the functions of missing isozymes. In fact, the *ss3a* mutant showed increased levels of SSI and GBSSI proteins, and these effects were clearly reflected in the starch structure (Fujita et al. 2007). Homozygous *ss1 ss3a* double mutant is sterile; however, genotypes heterozygous for one of mutant alleles exhibit starch accumulation (Fujita et al. 2011).

Active-type SSIIa derived from *indica* rice was introduced into the *ss1* and *ss3a* single mutant lines of *japonica* rice to generate *ss1 SS2a SS3a* and *SS1 SS2a ss3a* mutants, and their amylopectin structures were investigated. The results showed that rice lines with the *SS2a* allele, such as *ss1 SS2a SS3a* and *SS1 SS2a ss3a*, showed a reduction in amylopectin chains with DP ≤ 11 and an increase in amylopectin chains with 12 ≤ DP ≤ 24 compared with lines harboring the *ss2a*^*L*^ allele, such as *ss1 ss2a*^*L*^* SS3a* and *SS1 ss2a*^*L*^* ss3a*. These results indicate that SSIIa functions in the absence of SSI or SSIIIa (Crofts et al. 2017a).

**Fig. 3:**
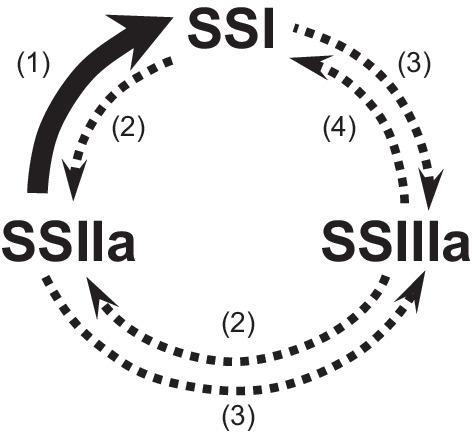
Summary of the relationships between three starch synthase (SS) isozymes in the rice endosperm. (1) Active SSIIa compensates for most of the function of SSI, particularly elongating amylopectin chains from the degree of polymerization (DP) of 6–7 to DP 8–12 (thick arrow). (2) The function of SSIIa could not be complemented by SSI or SSIIIa (dotted arrows). (3) Neither SSI nor SSIIa could compensate for the function of SSIIIa, which synthesizes amylopectin chains with DP > 35 (dotted arrows). (4) The function of SSI could not be complemented by SSIIIa (dotted arrow). This figure was obtained from Crofts et al. (2017a)

Analysis of *japonica ss1* or *ss3a* mutant rice revealed that each SS isozyme plays a distinct role in amylopectin biosynthesis. SSI elongates the non-reducing ends of amylopectin chains with DP 6 or 7 to DP 8–12 (Fujita et al. 2006); SSIIa functions to elongate amylopectin chains with DP 6–12 to DP 13–24 (Nakamura et al. 2005); and SSIIIa synthesizes long amylopectin chains with DP > 30 (Fujita et al. 2006, 2007). Generation of new rice lines by introducing *indica* rice-derived active-type *SS2a* allele into *japonica ss1* or *ss3a* mutant rice enabled the demonstration of compensatory roles among SSI, SSIIa, and SSIIIa (Crofts et al. 2017a). The chain-length distribution pattern of *indica* rice (*SS1 SS2a SS3a*) was similar to that of the *ss1 SS2a SS3a* line, containing active-type SSIIa in the absence of SSI. The absolute value of the subtraction curve of “*ss1 SS2a SS3a* (rice containing active-type SSIIa in the absence of SSI)—*SS1 SS2a SS3a* (wild-type *indica* rice)” was considerably lower than that of “*ss1 ss2a*^*L*^* SS3a* (*japonica ss1* mutant)—*SS1 ss2a*^*L*^* SS3a* (wild-type *japonica* rice)”. These results indicated that the active-type SSIIa enzyme derived from *indica* rice compensated most of the functions of SSI. The increase in the amount of amylopectin chains with DP 6–7 and the reduction in that of chains with DP 8–12 in the absolute value of the subtraction curve “*ss1 SS2a SS3a—SS1 SS2a SS3a*” were minimized by 80%; however, the increase of DP 16–19 was minimized by only 50%. This implies that SSIIa derived from *indica* rice compensates the function of SSI in elongating the non-reducing ends of A-chains with DP 6–7 into those with DP 8–12, but does not perfectly compensate its role in the elongation of the non-reducing ends of B_1_-chains into those with DP 16–19. By contrast, the *SS1 SS2a ss3a* line generated by introducing *indica* rice-derived active-type *SS2a* allele into the *ss3a japonica* mutant did not increase the amount of long amylopectin chains with DP > 30. This clearly indicates that SSIIa derived from *indica* rice does not compensate for the function of SSIIIa. Similarly, SSI could not compensate for the function of SSIIa or SSIIIa, and SSIIIa could not compensate for the function of SSI or SSIIa (Fig. 3; Crofts et al. 2017a).

## Introduction of *SS2a *and/or *GBSS1 *from *Indica *Rice into *ss3a ss4b*

SS enzymes possess a total of 11 isozymes: one SSI isozyme, three SSII isozymes, two SSIII isozymes, two SSIV isozymes, one SSV isozyme, and two GBSS isozymes. The function of each SS isozyme, including SSI, SSIIa, SSIIIa, and GBSSI, strongly expressed in the endosperm, became apparent through the characterization of the corresponding mutants and the biochemical analysis of purified enzymes. However, the functions of other SS isozymes have not yet been elucidated because they are expressed in tissues other than the endosperm. Mutants lacking SSIVb, one of the SSIV isozymes, have been isolated; however, their amylopectin structure and seed phenotype were the same as those of the wild-type. In contrast to the *ss4b* single mutant, double mutants lacking both SSIIIa and SSIVb (*ss2a*^*L*^* ss3a ss4b*) showed a remarkably different starch granule morphology (spherical) compared with the wild-type (polygonal) (Toyosawa et al. 2016). The amounts of B_2_ and B_3_ long amylopectin chains, which connect amylopectin clusters, were further reduced in *ss3a ss4b* mutant compared with *ss3a*. This suggests that SSIVb is involved in the synthesis of B_2_ and B_3_ chains of amylopectin, and therefore is functionally redundant to SSIIIa. Each amyloplast contains multiple starch granules, which form a compound structure in rice (Matsushima et al. 2013), within which individual starch granules are separated from each other by a septum-like structure (Kawagoe 2013). Immuno-electron microscopy observation using anti-SSIVb antibody revealed that SSIVb localizes to septum-like structures in the amyloplast in the developing endosperm of rice (Toyosawa et al. 2016), while SSIIIa localizes to the starch granules as well as the outer envelope membrane surrounding the amyloplast. Loss of SSIIIa alone results in slightly rounded starch granule morphology compared with the wild-type (Fujita et al. 2007). Confocal laser scanning microscopy analysis of the *ss3a ss4b* double mutant revealed the presence of compound-type starch granules, although they resembled the spherical simple-type starch granules in shape. Thus, the loss of both SSIIIa and SSIVb made the septum-like structure fragile, reduced starch biosynthesis, and fully expanded the starch granules, resulting in spherical starch granules (Toyosawa et al. 2016).

**Fig. 4:**
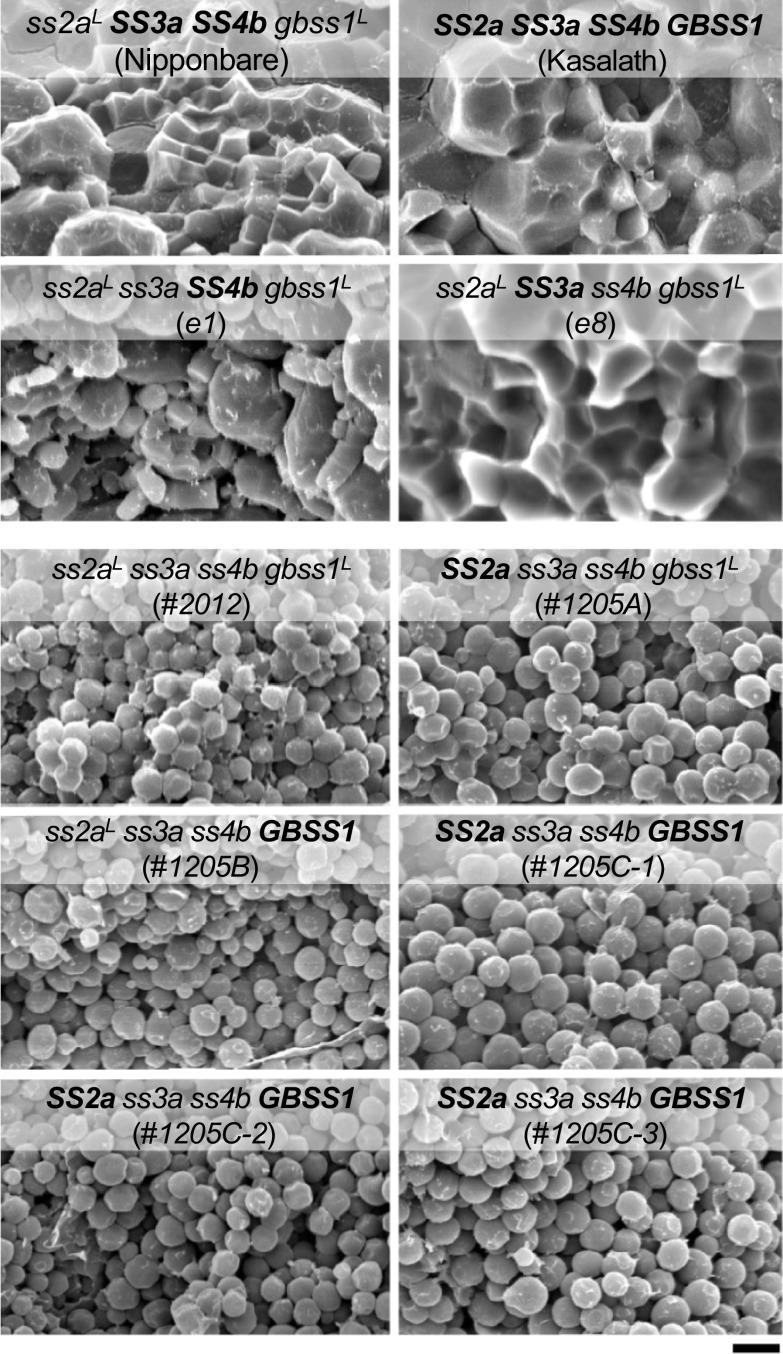
Scanning electron micrographs of the cross-sections of mature seeds. Scale bar = 5 μm. Data were obtained from Crofts et al. (2021b)

The *ss3a ss4b* mutants were derived from *japonica* rice, and therefore showed low SSIIa activity and GBSSI protein levels compared with *indica* rice (Nakamura et al. 2005; Sano 1984). To determine whether the alteration of starch granule morphology in *japonica ss3a ss4b* mutants could be complemented by SSIIa and/or GBSSI derived from *indica* rice, the corresponding genes were introduced into *ss2a*^*L*^* ss3a ss4b gbss1*^*L*^ by crossing with *indica* rice (*SS2a SS3a SS4b GBSS1*), and *SS2a ss3a ss4b gbss1*^*L*^, *ss2a*^*L*^* ss3a ss4b GBSS1*, and *SS2a ss3a ss4b GBSSI* were generated (Crofts et al. 2021a). However, the expression of *SS2a* and/or *GBSS1* in the *japonica ss3a ss4b* mutant did not recover the starch granule morphology from spherical to polygonal (Fig. 4). This implies that the loss of SSIIIa and SSIVb cannot be complemented by SSIIa or GBSSI (Crofts et al. 2021a). Lines containing active-type SSIIa, such as *SS2a ss3a ss4b gbss1*^*L*^ and *SS2a ss3a ss4b GBSS1*, showed lower levels of amylopectin chains with DP 7–12 and higher levels of those with DP ≥ 13 compared to the lines containing less active SSIIa. Amylose contents of *ss2a*^*L*^* ss3a ss4b GBSS1*, *SS2a ss3a ss4b GBSS1*, and *SS2a ss3a ss4b gbss1*^*L*^ were 40%, 36%, and 31%, respectively. The ADP-glucose content of the crude extract prepared from the developing endosperm of lines carrying the *gbss1*^*L*^ allele but lacking SSIIIa and SSIVb (such as *ss2a*^*L*^* ss3a ss4b gbss1*^*L*^ and *SS2a ss3a ss4b gbss1*^*L*^) was high, whereas that of lines with the *GBSS1* allele was low, perhaps because ADP-glucose was used for amylose synthesis (Crofts et al. 2021a).

## Introduction of *SS2a *and/or *GBSS1 *from *Indica* Rice into High RS Mutant Rice

As a potential target locus for increasing RS content in *indica* rice, a loss of function mutation in SSIIIa in the presence of *GBSS1* (*Wx*^*a*^) allele was identified by map-based cloning (Zhou et al. 2016). The rice grains of *ss3a GBSS1* had high amylose content (approximately 35%) and more amylose–lipid complex, which increased the RS content to 6% compared to that of the control line (approximately 1%) (Zhou et al. 2016). In comparison to the loss of SSIIIa, the loss of BEIIb drastically increases the RS content of endosperm starch (Tsuiki et al. 2016), as described above. This is because a considerable decline in the amount of short amylopectin branches proportionally increases the amount of long amylopectin chains, and a reduction in amylopectin biosynthesis elevates the amylose content; both of these factors contribute to the increase in RS content (Tsuiki et al. 2016; Zhou et al. 2016; Chen et al. 2017). To determine whether RS content can be further increased, *SS2a* and/or *GBSS1* alleles from *indica* rice were introduced into *japonica be2b* single mutant or *ss3a be2b* double mutant (Itoh et al. 2017; Miura et al. submitted). To practically utilize high RS rice lines, it is important to increase their yield. However, elevation of the RS content of the endosperm and increase in seed weight and yield are often a trade-off. To solve this problem, RS-rich rice lines were backcrossed three times with the high-yielding and large grain bearing rice cultivar, ‘Akita 63’ (Makino et al. 2020).

Compared with mutants lacking SS isozymes such as SSI, SSIIIa (Crofts et al. 2017a), or SSIIIa and SSIVb (Crofts et al. 2021a) described above, *japonica be2b* single mutant or *ss3a be2b* double mutant showed no significant change in starch structure upon the introduction of the *indica SS2a* allele (Itoh et al. 2017; Luo et al. 2020). This is because the number of short amylopectin branches (DP < 12), which serve as primers for SSIIa, was greatly reduced in the absence of BEIIb. On the other hand, introduction of the *GBSS1* allele into the *japonica be2b* single mutant increased the apparent amylose content to approximately 38% (equivalent to a 1.75-fold increase relative to the original *japonica be2b* mutant; Miura et al. submitted). Analyses of the recombinant inbred lines, which possess *SS2a* and *GBSS1* but lack BEIIb, also showed high amylose content (31–34%) but had some short branches on amylose (Zhang et al. 2022). Loss of SSIIIa is known to increase the apparent amylose content of *japonica* rice (*ss2a*^*L*^* ss3a be2b*) compared with wild-type and *be2b* single mutant *japonica* rice, because of a concomitant increase in GBSSI and AGPase protein levels (Asai et al. 2014). However, introduction of the *GBSS1* allele into the *japonica ss3a be2b* double mutant did not cause any further increase in the apparent amylose content (Miura et al. submitted), presumably because the ADP-glucose content reached its upper limit and was insufficient to meet the demand of increased GBSSI levels. The RS content of rice lines lacking BEIIb was higher than that of lines lacking SSIIIa and BEIIb, regardless of the presence or absence of *SS2a* and/or *GBSS1* alleles. This is because the loss of SSIIIa increases the expression levels of *SS1*, thus increasing the number of short amylopectin branches and decreasing the number of long amylopectin chains connecting amylopectin clusters (Fujita et al. 2007). Introduction of *SS2a* and/or *GBSS1* elevated the RS content, and backcrossing the resultant RS-rich lines increased their seed weight by up to 1.9-fold. This is likely because flowering time was advanced, and temperature during the seed development period became more suitable for efficient starch biosynthesis. The *ss2a*^*L*^* be2b GBSS1* rice line showed high RS content and outstanding seed weight (29 mg per grain) compared with typical *japonica* rice (20 mg per grain), indicating its potential for practical applications.

## Introduction of *SS2a *and/or *GBSS1 *from *Indica *Rice into *isa1*

In addition to elongation of glucans by SS and generation of branches by BE, the trimming of inappropriate glucan branches by one of the DBEs, called isoamylase 1 (ISA1), is essential for formulating properly structured insoluble starch and for generating plump seeds. Loss of ISA1 leads to the accumulation of water-soluble phytoglycogen and the production of shriveled seeds in many plant species, including maize (James et al. 1995) and rice (Nakamura et al. 1996, 1997; Kubo et al. 1999), as shown by the analyses of *isa1* (*sugary-1*, *sug-1*) mutants (Nakamura 2002). *Japonica isa1* mutants can be roughly divided into two groups: severe-type, with phytoglycogen accumulation in the entire endosperm; and mild-type, showing phytoglycogen accumulation in the seed core but starch accumulation in the seed periphery, which can be stained by iodine (Nakamura et al. 1997; Kubo et al. 1999). These differences are dependent on the site of mutation within the *ISA1* gene, but their exact cause remains unknown. Introduction of the *SS2a* allele from *indica* rice into severe-type *japonica isa1* mutant (EM914) using the transgenic approach resulted in the accumulation of iodine-stainable insoluble α-glucans in the endosperm (Fujita et al. 2012). This insoluble glucan contained longer short amylopectin branches than phytoglycogen, but showed no increase in the amount of long amylopectin chains (DP > 30). X-ray diffraction analyses revealed that this glucan showed weak B-type crystallinity, unlike phytoglycogen (no crystallinity) and wild-type rice (clear A-type crystallinity).

**Fig. 5:**
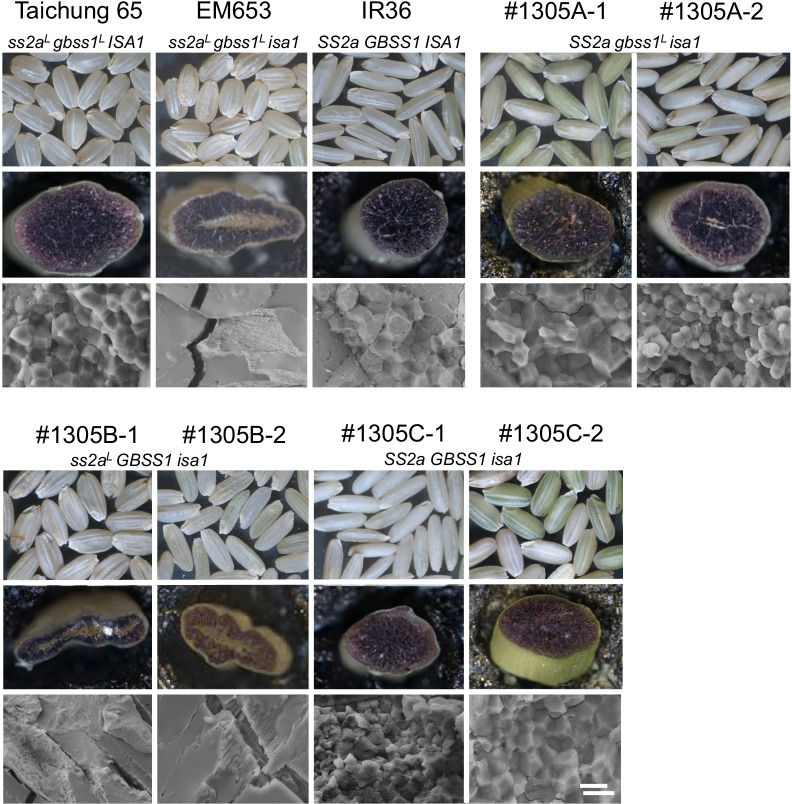
Analysis of rice seed morphology. Images show the morphologies of dehulled mature rice seeds (top), stereo-micrographs of iodine-stained cross-section of mature seeds (middle), and scanning electron micrographs of the central region of mature seeds (bottom). Data were obtained from Crofts et al. (2021a)

When *SS2a* and/or *GBSS1* alleles were introduced into the mild-type *japonica isa1* mutant (EM653) by conventional crossing, the resultant genotypes (*SS2a gbss1*^*L*^* isa1* and *SS2a GBSS1 isa1*) accumulated starch instead of phytoglycogen and produced plump seeds (Crofts et al. 2021b). While introduction of the *GBSS1* allele alone into the mild-type *japonica isa1* mutant (EM653) was phenotypically similar to EM653 and accumulated phytoglycogen in the central region of the shriveled seeds (Fig. 5). Amylopectin branches in rice lines harboring the *SS2a* allele (*SS2a gbss1*^*L*^* isa1* and *SS2a GBSSI isa1*) were elongated, and their chain-length distribution patterns (DP < 24) were shifted toward longer amylopectin chains, as in the severe-type *isa1 japonica* mutant transformed with the *SS2a* allele. The mild- and severe-type *isa1* mutants carrying the *SS2a* allele differed from each other in the amounts of long amylopectin chains (DP 30–60), which were higher in mild-type than in severe-type *isa1* mutant background, although the amounts of these amylopectin chains in *SS2a gbss1*^*L*^* isa1* and *SS2a GBSS1 isa1* lines were less than those in the wild-type. In addition, introduction of the *SS2a* allele into the mild-type *japonica isa1* mutant resulted in displaying clear A-type crystallinity and polygonal starch granules, similar to the wild-type; however, this result was not obtained by the introduction of *SS2a* into the severe-type *japonica isa1* mutant (Fujita et al. 2012).

Taken together, these findings suggest that SSIIa partially complements the loss of ISA1 by avoiding generation of phytoglycogen and by accumulating amylopectin. SSIIa could also sufficiently extend the branches of phytoglycogen. On the other hand, while the introduction of the *GBSS1* allele alone into the *japonica isa1* mutant could elevate the amylose content by 10%, it could not convert phytoglycogen into amylopectin, and the seeds remained shriveled.

## Practical Applications of Mutant Lines

The absence of enzymes required for starch biosynthesis partially inhibits the production of starch in the developing endosperm, leading to smaller grains and poor yield; the loss of BEIIb is a classic example. In addition, loss of more than one enzyme, such as both SSI and SSIIIa (Fujita et al. 2011) or both SSI and BEIIb (Abe et al. 2014), results in sterility. Unless the plant is completely sterile, the reduction in grain yield can be overcome by the introduction of loci responsible for high yield. As an attempt to improve agricultural traits, such as early flowering, large grain size, and high grain yield, we crossed rice mutants accumulating structurally unique starch with a high-yielding and elite rice cultivar ‘Akita 63’ (Makino et al. 2020).

The *ss3a* mutant accumulates a large amount of amylose, with almost no reduction in seed weight, compared with the wild-type; however, its late-flowering phenotype, as observed in our research paddy filed at high latitude in Akita prefecture, suggests that *ss3a* is not suitable for commercial cultivation. The *ss3a* mutant exhibited delayed flowering because its genes controlling flowering time were derived from the late-flowering parental rice cultivar ‘Nipponbare’. We backcrossed the *ss3a* mutant three times either with a high-yielding elite rice cultivar ‘Akita 63’ or with an excellent tasting elite rice cultivar ‘Akita Komachi’, resulting in the production of ‘Akita Sarari’ or ‘Akita Parari’, respectively (Fujita 2020). Cultivars ‘Akita 63’ and ‘Akita Komachi’ were selected as recurrent parents for backcrossing, since both these cultivars are suitable for cultivation in high latitude regions such as the Akita prefecture. In theory, genes derived from an elite parental rice cultivar account for 93.75% of the genetic background of the BC_3_ progeny. The flowering time of newly generated rice cultivars ‘Akita Sarari’ and ‘Akita Parari’ was similar to that of the elite rice cultivars (early August) and approximately 1 month earlier than that of the original *ss3a* mutant. The seed weight and yield of ‘Akita Sarari’ and ‘Akita Parari’ were also similar to those of ‘Akita 63’ and ‘Akita Komachi’, respectively. ‘Akita Sarari’ presented high yield and high amylose content, and its flour could be used for the production of noodles and bread. ‘Akita Parari’ exhibited a less sticky texture relative to the typical *japonica* rice cultivars and could be used for making pilaf, fried rice, and risotto. Both ‘Akita Sarari’ and ‘Akita Parari’ are now commercially available (https://starchtec.com/).

As described earlier, RS content and seed weight are often in a trade-off relationship in the absence of BEIIb. We tried to overcome this problem by crossing the high RS lines with a highly yielding rice cultivar. The high RS rice cultivar ‘Manpuku Surari’ was generated by backcrossing the *ss3a be2b* mutant (Asai et al. 2014) with ‘Akita 63’ three times. The resultant rice line ‘Manpuku Surari’ exhibited 1.5-fold higher seed weight compared with the original *ss3a be2b* mutant, and tenfold higher RS content in cooked rice and rice flour relative to the typical *japonica* rice, in addition to the ability to suppress postprandial increase in glucose levels (Saito et al. 2020). Thus, ‘Manpuku Surari’ is anticipated to be sold as “Foods with Function Claims”, as designated by the Consumer Affairs Agency.

Another effective way to overcome the low seed weight in high RS lines is to combine the absence of BEIIb with an absence of ISA1. ‘Chikushi-kona 85’ is a high RS *japonica*-rice mutant lacking BEIIb activity, but generated relatively plump seeds unlike shrunken seeds often seen in *be2b* mutants (Wada et al. 2018). The detailed genetic analyses of ‘Chikushi-kona 85’ and its parental mutant EM129 revealed that they were the double mutants lacking both BEIIb and ISA1 (Nagamatsu et al. 2022). BEIIb and ISA1 are the enzymes responsible for the generation of short amylopectin branches (Nishi et al. 2001; Tanaka et al. 2004) and removal of improper amylopectin branches (Nakamura 2002), respectively. The absence of these enzymes gives opposite effects on amylopectin structure. Loss of BEIIb drastically decreases short amylopectin chains with DP < 14 and increases long amylopectin chains (Nishi et al. 2001), while loss of ISA1 greatly increases short amylopectin chains with DP < 11 and decreases intermediate amylopectin chains (Nakamura et al. 1997). Both of these single mutant lines display shrunken or wrinkled seed phenotype with low seed weight (Nishi et al. 2001; Nakamura et al. 1997). Endosperm starch of ‘Chikushi-kona 85’ and EM129, both of which lack BEIIb and ISA1, showed milder starch phenotypes compared with the *be2b* single mutant, and accumulated a very small amount of phytoglycogen at the center of the seeds (Nagamatsu et al. 2022). In other words, the deficiency of BEIIb and ISA1 enzymes canceled each other's effects, and their double mutant resulted in high RS content and high seed weight (Nagamatsu et al. 2022). ‘Chikushi-kona 85’, generated by crossing EM129 with a high yield elite rice cultivar, was registered as a rice variety that suppresses fluctuation of postprandial blood glucose levels (Wada et al. 2018).

## Conclusions

Our results showed that the structure and properties of starch in the rice endosperm could be manipulated by abolishing and/or increasing the expression levels of various starch biosynthetic enzymes; however, the degree and type of change relative to wild-type rice varied widely. The longer the length between the non-reducing ends to the branch point of amylopectin branches within a cluster, the higher the temperature required for starch gelatinization. Moreover, as the gelatinization temperature increases, the retrogradation of starch becomes easier, while its degradation by digestive enzymes becomes more difficult. Conversely, the shorter the amylopectin branches, the easier the gelatinization and degradation, and the more difficult the retrogradation. Among the dozens of isozymes of starch biosynthetic enzymes, ISA1, SSIIa, and BEIIb had the greatest influence on the structure and properties of amylopectin. The length of amylopectin branches decreased in the absence of ISA1 and SSIIa, and increased in the absence of BEIIb, thus demonstrating that ISA1, SSIIa, and BEIIb directly influence the physicochemical properties of starch, including gelatinization temperature, retrogradation, and enzymatic degradation.

Research has also elucidated the order of starch biosynthesis. A recent study showed that BEIIb generates amylopectin branches near the amorphous region of the crystalline lamella (Nakamura et al. 2020). Amylopectin branches generated by BEIIb are first elongated by SSI by the addition of two glucose residues, and then further elongated by active-type SSIIa in *indica* rice (Abe et al. 2014; Crofts et al. 2017a). This is supported by the drastic reduction in SSI activity upon the loss of BEIIb (Nishi et al. 2001). Furthermore, SSI, SSIIa, and BEIIb are thought to form a trimeric complex, which facilitates efficient starch biosynthesis during seed development (Liu et al. 2012; Crofts et al. 2017b).

High-level expression of GBSSI in *japonica* rice mutants, achieved by the introduction of the *GBSS1* allele from *indica* rice, generally increases their amylose content. However, introduction of the *GBSS1* allele into the *ss3a be2b* double mutant, in which the expression level of the endogenous *GBSSI* gene is also high, does not cause any further increase in amylose content, most likely because of the depletion of its substrate (ADP-glucose) (Miura et al. submitted).

The generation of new allelic combinations of starch biosynthetic genes exhibiting no and/or high expression levels in rice, and analysis of their starch properties are starting to allow us to freely design the starch structure according to the application-based requirements. We have isolated numerous *japonica* rice mutant lines lacking starch biosynthetic enzymes, analyzed their starch phenotypes, and revealed the function of individual enzymes (Fujita 2014). However, a considerable proportion of the world’s population consumes *indica* rice as their staples. Comparing with the rich source of *japonica* rice mutant panels, the number of mutants isolated from *indica* rice and their reports are currently limited. However, presence of unique allele(s) that affect starch properties in *indica* rice is expected, such as *ALK*^*d*^ described in the present review article. In fact, six starch biosynthetic enzymes, SSI, SSIIa, GBSSI, BEI, BEIIa, and BEIIb, are known to have different alleles between *japonica* and *indica* rice cultivars. Their recombinant inbred lines showed that not only SSIIa but also SSI, EBI, and BEIIa gave a minor effect on starch structure (Luo et al. 2015). These suggest that the presence of unique alleles derived from *indica* rice that are different from the japonica rice. Accumulating the knowledge of different alleles in *indica* rice variety and combining with the alleles from japonica rice will enable to expand the variation of starch further. If these allelic combinations inhibit starch biosynthesis and deteriorate certain agricultural traits such as seed size, then the lines carrying these allelic combinations could be backcrossed with high-yielding elite varieties. Designing starch structures, breeding and cultivating to suit their growth environment and increase yield, and popularizing them will enable the development of a new type of rice starch, which can be used in a wide range of applications, and can contribute to the food industry and agricultural sector in the near future.

## Data Availability

Not applicable.

## Abbreviations

AGPase: ADP-glucose pyrophosphorylase
BE: Starch branching enzymes
DBE: Starch debranching enzymes
DP: Degree of polymerization
GBSS: Granule-bound starch synthase
ISA: Isoamylase
RS: Resistant starch
SNP: Single nucleotide polymorphism
SS: Starch synthases
